# Variants in the *FFAR1* Gene Are Associated with Beta Cell Function

**DOI:** 10.1371/journal.pone.0001090

**Published:** 2007-11-07

**Authors:** Martins Kalis, Per Levéen, Valeriya Lyssenko, Peter Almgren, Leif Groop, Corrado M. Cilio

**Affiliations:** 1 Department of Clinical Sciences, Cellular Autoimmunity Unit, Lund University, Malmö University Hospital, Malmö, Sweden; 2 Department of Clinical Sciences, Diabetes and Endocrinology, Lund University, Malmö University Hospital, Malmö, Sweden; University of California at Los Angeles, United States of America

## Abstract

**Background:**

The FFAR1 receptor is expressed mainly in pancreatic beta cells and is activated by medium to long chain free fatty acids (FFAs), as well as by thiazolidinediones, resulting in elevated Ca^2+^ concentrations and promotion of insulin secretion. These properties suggest that FFAR1 could be a mediator of lipotoxicity and a potential candidate gene for Type 2 diabetes (T2D). We therefore investigated whether variations at the *FFAR1* locus are associated with T2D and beta cell function.

**Methodology/Principal Findings:**

We re-sequenced the *FFAR1* region in 96 subjects (48 healthy and 48 T2D individuals) and found 13 single nucleotide polymorphisms (SNPs) 8 of which were not previously described. Two SNPs located in the upstream region of the *FFAR1* gene (rs1978013 and rs1978014) were chosen and genotyped in 1929 patients with T2D and 1405 healthy control subjects. We observed an association of rs1978013 and rs1978014 with insulinogenic index in males (p = 0.024) and females (p = 0.032), respectively. After Bonferroni corrections, no association with T2D was found in the case-control material, however a haplotype consisting of the T-G alleles conferred protection against T2D (p = 0.0010).

**Conclusions/Significance:**

Variation in the *FFAR1* gene may contribute to impaired beta cell function in T2D.

## Introduction

Deterioration of beta cell function is a hallmark of Type 2 diabetes (T2D) and is considered to contribute to worsening of glucose tolerance with time [1]–[4]. While the underlying causes are not fully understood, chronic exposure of beta cells to high concentrations of glucose (glucotoxicity) and free fatty acids (FFAs) (lipotoxicicity) have been suggested to induce irreversible changes in islet function [5]–[7]. The pathways by which glucose enters into beta cells are well described but less is known by which mechanisms elevated FFAs might affect beta cells and their function.

The free fatty acid receptor FFAR1 (GPR40–G protein-coupled receptor 40) is the first gene product identified to act as an extracellular membrane receptor for FFAs. It was recently shown to be activated in pancreatic beta cells *in vitro* by medium to long chain free fatty acids (FFAs) [8]–[10] as well as by thiazolidinediones (Rosiglitazone and MCC-555) [9], causing elevated Ca^2+^ concentrations and subsequent promotion of insulin secretion. *FFAR1* is located in the 19q13.1 chromosomal region, which has been linked to T2D [11], [12] and T2D-related phenotypes [4], [13] in several genome wide scans. The open reading frame of the gene encompasses a single exon of 903 bp encoding seven transmembrane domains, characteristic of G protein-coupled receptors (GPCRs) [14]. Studies in rodents and humans have shown that the FFAR1 is expressed mainly in pancreatic beta cells, but also in the brain [8]–[10]. Mice with overexpression of FFAR1 show impaired beta cell function and develop diabetes, whereas disruption of the gene reduces FFA-stimulated insulin release [15] and, according to Steneberg et al., protects from diabetes [16]. These properties make FFAR1 an interesting candidate for mediating lipotoxicity in beta cells although its role in this process is not fully unravelled. To investigate whether variation in the *FFAR1* locus is associated with human T2D, we re-sequenced the *FFAR1* gene and tested two of the identified SNPs for association with T2D and beta cell function.

## Materials and Methods

### Study subjects

To identify SNPs in the *FFAR1* region, 48 T2D patients (31 male, 17 females, age 51±6, BMI 25.4±1.7) and 48 healthy glucose-tolerant control subjects (20 males, 28 females, age 62±7, BMI 23.2±2.1) from the Botnia study [17] were selected for initial sequence analysis.

The case-control material genotyped consisted of 1929 patients with T2D and 1405 control subjects from Finland and Sweden (Table 1). In addition, we studied whether SNPs in the *FFAR1* gene influenced insulin secretion and FFA levels measured during oral glucose tolerance test (OGTT) in 1011 non-diabetic individuals participating in the Botnia study (Table 1) [17]. Study subjects were unrelated except 354 sibling pairs (each pair from different family) included in the cohort of 1011 healthy individuals.

**Table 1:** Clinical characteristics of patient samples

	Association study		Healthy Botnia individuals
	T2D	CONTROLS	
*n*	1929*	1405**	1011
Sex (males/females)	1086/843	652/753	475/536
Age (years)	61.6±11.5	52.8±13.6	50±15
Age of onset of T2D (years)	56.7±11.4		
BMI (kg/m^2^)	29.58±5.33	25.92±3.78	26.8±4.2
Fasting plasma glucose (mmol/l)	11.36±4.19	5.38±0.44	5.59±0.58
2 hour plasma glucose (mmol/l)	15.54±5.7	5.67±1.10	6.47±1.63
C-peptide (nmol/l)	1.03±0.55	ND	ND
Fasting plasma insulin (pmol/l)	69.32±61.01	40.79±24.27	50.95±29.95
2 hour insulin (pmol/l)	354.45±323.46	205.85±165.44	300.22±262.66
HOMA	5.80±8.51	1.58±0.97	2.15±1.38
Insulinogenic index	2.84±3.77	5.86±4.17	5.84±3.98
HbA_1c_ (%)	7.73±2	5.19±0.5	5.44±0.49
Triglicerides (mmol/l)	2.4±2.27	1.29±0.76	1.38±0.89
Cholesterol (mmol/l)	5.58±1.29	5.83±1.18	5.67±1.12
HDL cholesterol (mmol/l)	1.16±0.32	1.37±0.41	1.37±0.34
Systolic blood pressure (mmHg)	144.55±22.05	133.48±19.22	132.78±19.74
Diastolic blood pressure (mmHg)	81.06±10.87	81.21±10.57	80.51±10.27

Legend to Table 1. Data are mean±SD. ND: not determined.

* 1455 T2D patients were measured for C peptide but the rest 451–for fasting plasma insulin (HOMA index was calculated from these). Values of 2 hrs glucose and insulin were obtained for 256 and 251 individual, respectively. Insulinogenic index could be calculated for 192 T2D patients. Blood pressure information was obtained from 1438 patients.

** Values of 2 hrs glucose and insulin, HOMA, insulinogenic index and HDL cholesterol were available only in 68% of the control group but trigliceride values in 87% of the individuals.

Diagnosis of T2D was based according to WHO criteria [18], GADA negative status and age at onset >35 years (except for three individuals which were below 35 years). Weight, height, plasma glucose, plasma insulin, triglicerides, total cholesterol, HDL cholesterol and blood pressure were measured and oral glucose tolerance test (OGTT) performed as described by Groop et al. [17]; plasma FFA levels were determined using an enzymatic colorimetric method (NEFAC ACS-ACOD Method, Wako Chemicals, Richmond, VA). Haemoglobin A_1c_ (HbA1c) was measured by high-pressure liquid chromatography with a normal range of 4–6% (Diamat Analyzer, Biorad Laboratories, Germany). Body mass index (BMI) was calculated as weight (in kilograms) divided by height (in meters) squared. Insulinogenic index (Δ Insulin _30 min-fasting_/Glucose _30 min_) and homeostasis model assessment index (HOMA) [19] was used to describe insulin secretion and insulin resistance, respectively. All subjects gave their informed consent at the time of entering to the study, which was approved by local ethics committees at Helsinki and Lund Universities.

### Sequencing of *FFAR1*

A 2379 bp long DNA including the 903 bp of the coding region in the *FFAR1* gene was re-sequenced. Reference sequence was taken from the NCBI database (http://www.ncbi.nlm.nih.gov)[20]. In order to sequence the *FFAR1* region, four primer pairs of overlapping PCR products were designed using program Primer Premier 5.0 (Premier Biosoft International). Standard protocols for PCR were as follows (5′-3′ sequences of forward and reverse primers, product size, annealing temperature): 1) CTCCCCTTCCGGCTCACT, CTCTCCACCATGTCACCTCTTA, 566 bp, 50°C; 2) CAGGAGTCAAACTCCCATTCC, AGGTGTTGCTGTGGTCCAG, 901 bp, 62°C (designed by MRC Geneservice, Cambridge, UK); 3) TTGGGCTACCAAGCCTTC, CTGCAGTTCCTCCGAAGC, 658 bp, 60°C; 4) GTGACCGGTTACTTGGGAAG, CTTTGGGGGAGTCAAAGTCAT, 752 bp, 62°C. PCR reactions were performed on GeneAmp PCR System 9700 (Perkin Elmer).

Sequencing reactions were performed using BigDye v3.1 kit (Applied Biosystems) on GeneAmp PCR System 9700 according to manufacturers protocol. Sequencing products were purified with DyeEx 96 kit (QIAGEN) and run on ABI Prism 3730 Genetic Analyzer (Applied Biosystems). Sequencing data were analyzed with program Sequencher 3.1.2 (Gene Codes Corporation, Ann Arbor, MI, USA).

Selected SNPs were genotyped using the SNaPshot assay on an ABI 3100 DNA sequencer (Applied Biosystems) according to the manufacturer′s protocol.

### Genotyping

PCR fragment for the multiplex SNP genotyping of FFAR1 polymorphisms rs1978013 and rs1978014 with SNaPshot assay was amplified in 20 µl reaction volume at an annealing temperature of 55°C, primers (5′-3′): CTCCCCTTCCGGCTCACT and CTCTCCACCATGTCACCTCTTA. PCR products were treated with Shrimp Alkaline Phosphatase (SAP) (USB Corporation) and Exonuclease I (New England Biolabs). Extension primer sequence (5′-3′) for rs1978013 was ACCGTGACATGGATGGGGCC and for rs1978014–AACTGGGGAGAGCCCAAGGGTCAGC, where the underlined nucleotides indicate an unspecific primer tail included in order to produce differently sized products. The minisequencing reaction was performed on GeneAmp PCR System 9700 according to manufacturer's protocol. After SAP/Exonuclease I treatment, samples where run on ABI Prism 3100 Genetic Analyzer and data analyzed using the GeneMapper 3.1 software (Applied Biosystems).

### Statistical and bioinformatic analysis

Linkage disequilibrium (LD) calculations and haplotype analysis were carried out using Haploview 3.32 software (http://www.broad.mit.edu/mpg/haploview/)[21], [22]. For haplotype reconstruction and subsequent analysis of metabolic parameters, we used HAP haplotype analysis system available online at http://research.calit2.net/hap [23], [24]. Power calculations were performed online using Genetic Power Calculator (http://ibgwww.colorado.edu/pshaun/gpc)[25], [26]. Chi-squared test was used to compare the general genotype frequency distribution among T2D and control groups. Odds ratios were calculated using logistic regression analysis with age, BMI and sex as covariates. Mantel-Haenszel test was used to test for heterogeneity between the Swedish and Finnish samples. Differences in insulinogenic index and FFA levels between different genotype carriers were assessed using either ANOVA (for equal weight of genotypes) or linear regression analysis with age, BMI and sex as covariates. A robust variance estimator was used to adjust for the possible non-independence due to family clustering. Correlation between insulin secretion and FFA levels was analysed using multivariate regression correlation matrix (NCSS software). The standard errors were adjusted for repeated measurements made on the same individual by methods of GEE methodology. A p value of <0.05 was considered statistically significant. Bonferroni adjustments for multiple measurements were done were appropriate: p-values were corrected by four in the case-control study (two SNPs tested, stratified by sex), by eight in the phenotype analysis (two SNPs tested for insulinogenic index and post-OGTT FFA levels, stratified by sex) and by eight in the phenotype analysis in haplotypes (four haplotypes tested for insulinogenic index and post-OGTT FFA levels). We chose the additive model as the main hypothesis because it is the model that corresponds better to the putative biological function of FFAR1 receptor [27]–[29]. Therefore p values were not corrected for other genetic models explored (equal weight, recessive and dominant). To correct for multiple testing in the haplotype analysis, 10000 permutations were done using the Haploview software. The 5′ upstream region of the *FFAR1* gene was analysed for putative regulatory elements using the MatInspector program (http://www.genomatix.de/products/MatInspector/) [30], [31].

## Results

We first re-sequenced a 2379 bp fragment of the *FFAR1* region in a screening panel of 96 subjects (48 T2D and 48 healthy subjects). We identified 13 SNPs and two of these polymorphisms where located in the coding region of the gene, including one synonymous (Val) and one non-synonymous (Arg/His, rs2301151) polymorphism. Five of the SNPs (rs1978013, rs1978014, rs10418569, rs2301151 and rs1573611) were previously registered in the NCBI database [20], whereas eight SNPs are new (Figure S1).

There was no clear linkage disequilibrium between the 13 SNPs (Figure S2). The minor allele frequencies (MAF) of the eight newly discovered SNPs were less then 0.02, the other five SNPs had MAF 0.10–0.48.

Of them, only the frequencies of the two SNPs at positions 598 and 597 bp upstream of the open reading frame (rs1978013 and rs1978014; Figure S1) differed between the T2D and control subjects in the screening panel (rs1978013: 13 TT, 25 TC and 10 CC in T2D vs. 15 TT, 31 TC and 2 CC in controls, p = 0.047 and for rs1978014: 4 AA, 4 AG and 9 GG in T2D patients with BMI<30 vs. 12 AA, 20 AG and 7 GG in control individuals with BMI<30, p = 0.024). These two SNPs were then further tested for association with T2D in a case-control material from Finland and Sweden consisting of 1929 patients with T2D and 1405 control subjects. All genotypes were in Hardy-Weinberg equilibrium, both in cases and in controls.

The Tagger algorithm [32] of the Haploview software showed that with r^2^ threshold of 0.3 rs1978013 captures rs10418569 (D' 0.94, r^2^ 0.33) and rs1573611 (D' 0.89, r^2^ 0.31), whereas rs1978014 captures rs10418569 (D' 1, r^2^ 0.28) and rs2301151 (D' 1, r^2^ 0.12) at r^2^ threshold 0.1 (Figure S2).

The bioinformatic analysis revealed three potential transcription factor-binding sites in the vicinity of rs1978013 and rs1978014–ATF6 (located −16 to −10 bp or −17 to −11 bp upstream of rs1978013 and rs1978014, respectively), INSM1 (−11 to −3 bp/−12 to −4 bp) and NF-kappaB (+24 to +33 bp/+23 to +32 bp downstream).

There was more than 80% power assuming an additive model to detect an OR of 1.32 (p = 0.05) in the case-control material. However, after Bonferroni correction, no odds ratio was significantly associated with T2D. SNP rs1978014 had an OR 1.26 (95% CI 1.00–1.61) for association with T2D (uncorrected p = 0.049, corr. p = 0.196, Table 2), while CC genotype of rs1978013 was more frequent in T2D group only in males (T2D 17.9%, controls 15.5%, OR 1.49, 95% CI 1.07–2.07, uncorr. p = 0.019, corr. p = 0.076; Table S1).

**Table 2:** Genotype frequencies and odds ratios of the rs1978013 and rs1978014

Genotypes and alleles	T2D *n* (%) (n = 1929)	Controls *n* (%) (n = 1405)	P (χ^2^ test)	Additive model OR (95% CI)	p	Dominant model OR (95% CI)	p	Recessive model OR (95% CI)	p
rs1978013									
TT	621 (32.2)	503 (35.8)		1	-				
TC	969 (50.2)	673 (47.9)		1.09 (0.90–1.30)	0.317/NS				
CC	339 (17.6)	229 (16.3)	0.09/0.36	1.24 (0.97–1.58)	0.082/0.328	1.12 (0.94–1.33)	0.185/0.74	1.18 (0.95–1.47)	0.135/0.54
T	2211 (57.3)	1679 (59.8)							
**C**	1647(42.7)	1131 (40.2)	**0.046/**0.184						
rs1978014									
GG	327 (17)	286 (20.4)		1	-				
AG	910 (47.3)	676 (48.1)		1.13 (0.94–1.36)	0.204/0.816				
AA	686 (35.7)	443 (31.5)	**0.01/0.04**	1.27 (1.00–1.61)	**0.049/**0.196	1.18 (0.96–1.46)	0.116/0.464	1.16 (0.98–1.39)	0.085/0.34
G	1564 (40.7)	1248 (44.4)							
**A**	2282 (59.3)	1562 (55.6)	**0.002/0.008**						

Legend to Table 2. Logistic regression results are adjusted for age, sex, BMI and family dependence. Both nominal and the Bonferroni-corrected p-values are shown. NS: not significant.

Haplotype analysis revealed that a haplotype consisting of the T-G alleles (permutated p = 0.001) of SNPs rs1978013 and rs1978014 conferred protection whereas the C-A haplotype conferred increased risk (permutated p = 0.0486) of T2D (Table 3).

Post-OGTT FFA concentration did not significantly differ between different genotype carriers after Bonferroni corrections (Table 4, Table S2). Post-OGTT FFA concentration was lower in the CC/CT carriers (223±95 mmol/l) vs. the TT genotype (244±148 mmol/l) of rs1978013 (uncorr. p = 0.010, corr. p = 0.08, Table 4). After stratification for sex, post-OGTT FFA levels of rs1978013 were lower in males (TT = 268±189, TC = 239±96, CC = 215±102 mmol/l, uncorr. p = 0.012, corr. p = 0.096, Table S2). In addition, the T-G haplotype showed association with post-OGTT FFA levels which disappeared after Bonferroni correction (T-G 231±120 vs C-A 225±98, T-A 244±139, C-G 210±89 mmol/l, uncorr. p = 0.021, corr. p = 0.168, Table 5).

The rs1978013 was associated with insulinogenic index only in males (TT = 6.2±3.8, TC+CC = 5.3±3.4, uncorr. p = 0.003, corr. p = 0.024) while rs1978014 was associated with insulinogenic index only in females (GG+AG = 6.4±4.7, AA = 5.2±2.9, uncorr. p = 0.004, corr. p = 0.032; Table S2). In the whole sample, insulinogenic index was associated neither with genotypes (Table 4) nor haplotypes (Table 5) after Bonferroni corrections were done. Insulin secretion was lower in C allele carriers of rs1978013 (TT = 6.3±4.3, TC+CC = 5.6±3.8, uncorr. p = 0.010, corr. p = 0.08) and for AA genotype in rs1978014 (GG+AG = 6±4.2, AA = 5.3±3.2, uncorr. p = 0.030, corr. p = 0.24) (Table 4). The C-A haplotype had the lowest insulinogenic index (C-A 5.5±3.7 vs T-G 6.1±4.3, T-A 6±3.9, C-G 5.8±3.8, uncorr. p = 0.008, corr. p = 0.064, Table 5).

**Table 3:** Association of haplotypes with T2D

Allele		Haplotype frequency		p value	permutation p value *
rs1978013	rs1978014	T2D (n = 1929)	Controls (n = 1405)		
T	G	0.24	0.28	**0.0009**	**0.0010**
C	A	0.26	0.24	**0.0244**	**0.0486**
T	A	0.33	0.32	0.2537	0.5397
C	G	0.17	0.17	0.8663	0.9973

Legend to Table 3. * To correct for multiple testing, 10000 permutations were done.

**Table 4:** Insulinogenic index and post-OGTT FFA levels in different genotype carriers

	rs1978013			p	rs1978014			p
	TT (n = 327)	TC (n = 503)	CC (n = 181)		GG (n = 215)	AG (n = 510)	AA (n = 286)	
*Insulinogenic index*								
Equal weight	6.3±4.3	5.8±4	5.3±3.2	**0.022**/0.176	5.9±4	6.1±4.3	5.3±3.2	**0.030**/0.24
Dominant model	6.3±4.3	5.6±3.8		0.010*/0.08	5.9±4	5.8±4		0.892*/NS
Recessive model	6±4.1		5.3±3.2	0.154*/NS	6±4.2		5.3±3.2	0.030*/0.24
*2h FFA levels (mmol/l)*								
Equal weight	244±148	226±95	213±96	**0.008**/0.064	221±92	230±127	236±108	0.379/NS
Dominant model	244±148	223±95		0.010*/0.08	221±92	232±121		0.363*/NS
Recessive model	233±119		213±96	0.038*/0.304	227±118		236±108	0.519*/NS

Legend to Table 4. n = 1011. Data are mean±SD. Asterisk (*) indicates results from linear regression analysis, adjusted for age, sex, BMI and family dependence. Both nominal and the Bonferroni-corrected p-values are shown. NS: not significant.

**Table 5:** Insulinogenic index and post-OGTT FFA levels in rs1978013-rs1978014 haplotypes

Haplotype	n	Insulinogenic index	score	p	Post-OGTT FFA level (mmol/l)	score	p
T-G	697	6.1±4.3	0.076	0.121/0.968	231±120	−0.09	**0.021**/0.168
C-A	623	5.5±3.7	−0.095	**0.008**/0.064	225±98	0.04	0.763/NS
T-A	459	6.0±3.9	0.022	0.987/NS	244±139	0.026	0.969/NS
C-G	241	5.8±3.8	−0.006	1.000/NS	210±89	0.03	0.921/NS

Legend to Table 5. n = 1011. Data are mean±SD. Scores of the linear regression analysis are reported. Both nominal and the Bonferroni-corrected p-values are shown. NS: not significant.

## Discussion

The key finding of the present study was that rs1978013 and rs1978014 polymorphisms located upstream of the coding region of the *FFAR1* gene were associated with beta cell function in males and females respectively, however, due to the loss of statistical significance during Bonferroni corrections–not in the whole sample. Carriers of the CC genotype of rs1978013 had the lowest insulinogenic index and highest risk of T2D (although not significant after Bonferroni correction) but rs1978014 associates with T2D according to chi^2^ test. Increased T2D risk was discovered in the C-A haplotype carriers of SNPs rs1978013 and rs1978014.

The different association between the two polymorphisms in the *FFAR1* gene and insulin secretion in males and females might suggest that these polymorphisms may affect insulin sensitivity in a sex-specific manner in accordance with the finding of Hevener et al. showing that female rats are protected from lipid-induced reductions in insulin action [33]. We therefore tested this hypothesis by performing gender-specfic analyses of the putative effect of these SNPs on insulin sensitivity (HOMA). There was no association between HOMA and these polymorphisms, neither in the whole cohort nor after stratification for sex (data not shown). Correlation between HOMA and post-OGTT FFA level was also very weak (males: r^2^ = 0.17, p = 0.0002, females: r^2^ = 0.20, p = 0). This indicates that sex-specific differences in insulin sensitivity did not influence our results.

Although close to each other, rs1978013 and rs1978014 did not show any LD. This could possibly be explained by a recombination hot spot in this particular gene region. However, rs1978013 and rs1978014 captured the other common polymorphisms in the sequenced DNA region according to D' (Figure S2) at a low r^2^ threshold 0.1–0.3. In addition to association with T2D in the initial screening panel, lack of LD between the two neighbouring SNPs made these very close nucleotide positions of particular biological interest. The findings rather suggested that both SNPs, rs1978013 and rs1978014, together formed a haplotype, which increased risk of T2D.

FFAR1 was recently described as a cell-surface bound receptor for FFAs making it a candidate to mediate the negative effects of FFAs on beta cell function (lipotoxicity) [8]–[10]. However, two recent studies did not find an association between variations in the *FFAR1* coding region and the risk of developing T2D [34], [35]. Despite the lack of an association with T2D, Ogawa *et al*. found that the Arg211His polymorphism (rs2301151) in the coding region of *FFAR1* influenced serum insulin levels in 327 healthy Japanese males [34]. Although this polymorphism is in LD with rs1978013 and rs1978014 according to D' = 1, however the r^2^ is low (0.08 for rs1978013 and 0.12 for rs1978014) (Figure S2) suggesting an independent association of these two SNPs with insulin secretion.

We have also analysed whether any SNPs in the recent whole genome association study [36] would cover the *FFAR1* gene. Unfortunately this region was not covered by any informative SNPs on the Affymetrix chip. The 19q13 chromosomal region where *FFAR1* is situated has shown linkage with T2D as well as T2D and lipid-related phenotypes [11], [37]–[40]. However, we can only speculate whether the linkage could be attributed to *FFAR1* since the density of markers used in those studies is not high enough (approximate interval 7–13 cM) to pinpoint *FFAR1* gene as a strong candidate for the reported linkage.

Little is known about the promoter structure and transcription factor binding sites of the *FFAR1* gene. *FFAR1* lacks a canonical TATA box, consistent with reports of other TATA-less G protein-coupled receptors [41], [42]. The rs1978013 and rs1978014 polymorphisms apparently do not directly affect any potential transcription factor-binding motif. However, by sequence analysis, we identified three potential binding motifs for transcription factors which could be implicated in T2D development or beta cell function–ATF6 [43], INSM1 [44] and NF-kappaB [45] in close vicinity to rs1978013 and rs1978014. We can therefore not exclude the possibility that polymorphisms in the rs1978013 and rs1978014 might affect FFAR1 expression by altering transcription or they might be in linkage disequilibrium with another polymorphism affecting FFAR1 expression or function. Recently, Tomita et al. reported that insulinogenic index positively correlated with the *FFAR1* mRNA level in human pancreatic islets [46], however, whether variations in the *FFAR1* gene induce up- or down regulation of gene expression remains unknown. Identification of regulatory region and subsequent functional studies are necessary to understand the hypothetical effect of rs1978013 and rs1978014 variants on FFAR1 expression levels.

In summary, our study suggests an effect of polymorphisms in the FFAR1 gene on insulin secretion during an OGTT thereby making it a potential candidate to mediate lipotoxicicty in T2D.

## Supporting Information

Figure S1 The sequenced region with the detected SNP positions. A 2379 bp long DNA sequence including the 903 bp of the coding region (underlined) in the FFAR1. Five SNPs (framed-rs1978013, rs1978014, rs10418569, rs2301151 and rs1573611) were registered in the NCBI database before, whereas eight (highlited) were not. The rs1978013 and rs1978014 SNPs are located at position 208 and 209, respectively.
(0.06 MB TIF)

Figure S2 LD structure of the sequenced FFAR1 region. SNPs are numbered according to their position in the sequenced region, nucleotide position 208 and 209 correspond to rs1978013 and rs1978014, respectively, and positions 592, 1437 and 2059-to rs10418569, rs2301151 (Arg211His) and rs1573611, respectively.
(0.94 MB TIF)

Table S1 Sex-specific genotype frequencies and odds ratios of the rs1978013. Logistic regression results are adjusted for age, sex, BMI and family dependence. Both nominal and the Bonferroni-corrected p-values are shown. NS: not significant.
(0.04 MB DOC)

Table S2 Insulinogenic index and post-OGTT FFA levels in different genotype carriers stratified for sex. Males: n = 475, females: n = 536. Data are mean±SD. Asterisk (*) indicates results from linear regression analysis, adjusted for age, BMI and family dependence. Both nominal and the Bonferroni-corrected p-values are shown. NS: not significant.
(0.05 MB DOC)
