# Supplementary figures and images for: Variants in the FFAR1 Gene Are Associated with Beta Cell Function
Source: PLoS One. 2007 Nov 7;2(11):e1090. doi: 10.1371/journal.pone.0001090 (PMC2042513; doi:10.1371/journal.pone.0001090)

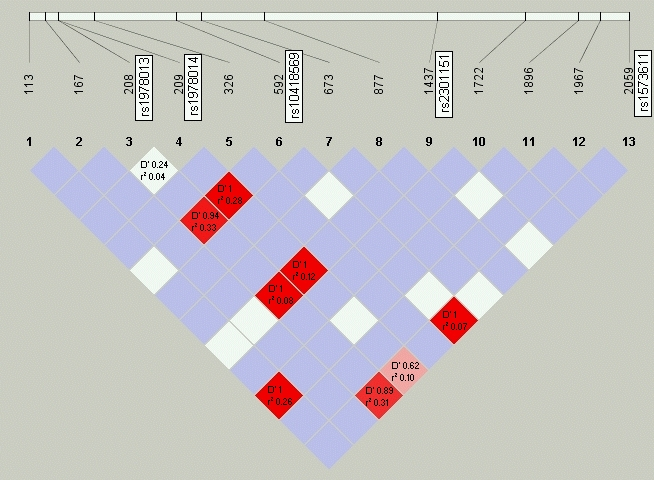

Supplement: Figure S2 — LD structure of the sequenced FFAR1 region. SNPs are numbered according to their position in the sequenced region, nucleotide position 208 and 209 correspond to rs1978013 and rs1978014, respectively, and positions 592, 1437 and 2059-to rs10418569, rs2301151 (Arg211His) and rs1573611, respectively. (0.94 MB TIF) [file pone.0001090.s002.tif]
