# Supplementary material for: Variants in the FFAR1 Gene Are Associated with Beta Cell Function
Source: PLoS One. 2007 Nov 7;2(11):e1090. doi: 10.1371/journal.pone.0001090 (PMC2042513; doi:10.1371/journal.pone.0001090)
Supplement: Table S1 — Sex-specific genotype frequencies and odds ratios of the rs1978013. Logistic regression results are adjusted for age, sex, BMI and family dependence. Both nominal and the Bonferroni-corrected p-values are shown. NS: not significant. (0.04 MB DOC) [file pone.0001090.s003.doc]

| Genotypes and alleles | T2D *n* (%) | Controls *n* (%) | p | Additive model | p | Dominant model | p | Recessive model | p |
| --- | --- | --- | --- | --- | --- | --- | --- | --- | --- |
|  |  |  | (chi^2^ test) | OR (95% CI) |  | OR (95% CI) |  | OR (95% CI) |  |
| Males | (n=1086) | (n=652) |  |  |  |  |  |  |  |
| TT | 356 (32.8) | 245 (37.6) |  | 1 | - |  |  |  |  |
| TC | 536 (49.4) | 306 (46.9) |  | 1.07 (0.84-1.37) | 0.583/NS |  |  |  |  |
| CC | 194 (17.9) | 101 (15.5) | 0.102/0.408 | 1.49 (1.07-2.07) | **0.019**/0.076 | 1.16 (0.92-1.47) | 0.198/0.792 | 1.43 (1.06-1.93) | **0.020**/0.080 |
|  |  |  |  |  |  |  |  |  |  |
| T | 1248 (57.5) | 796 (61.0) |  |  |  |  |  |  |  |
| **C** | 924 (42.5) | 508 (39.0) | **0.038**/0.152 |  |  |  |  |  |  |
|  |  |  |  |  |  |  |  |  |  |
| Females | (n=843) | (n=753) |  |  |  |  |  |  |  |
| TT | 265 (31.4) | 258 (34.3) |  | 1 | - |  |  |  |  |
| TC | 433 (51.4) | 367 (48.7) |  | 1.11 (0.85-1.45) | 0.435/NS |  |  |  |  |
| CC | 145 (17.2) | 128 (17.0) | 0.466/NS | 1.01 (0.70-1.45) | 0.957/NS | 1.09 (0.84-1.40) | 0.522/NS | 0.95 (0.68-1.31) | 0.737/NS |
|  |  |  |  |  |  |  |  |  |  |
| T | 963 (57.1) | 883 (58.6) |  |  |  |  |  |  |  |
| **C** | 723 (42.9) | 623 (41.4) | 0.387/NS |  |  |  |  |  |  |
