# Supplementary material for: Variants in the FFAR1 Gene Are Associated with Beta Cell Function
Source: PLoS One. 2007 Nov 7;2(11):e1090. doi: 10.1371/journal.pone.0001090 (PMC2042513; doi:10.1371/journal.pone.0001090)
Supplement: Table S2 — Insulinogenic index and post-OGTT FFA levels in different genotype carriers stratified for sex. Males: n = 475, females: n = 536. Data are mean±SD. Asterisk (*) indicates results from linear regression analysis, adjusted for age, BMI and family dependence. Both nominal and the Bonferroni-corrected p-values are shown. NS: not significant. (0.05 MB DOC) [file pone.0001090.s004.doc]

|  | rs1978013 | | | p |  | rs1978014 | | | p |
| --- | --- | --- | --- | --- | --- | --- | --- | --- | --- |
|  | TT | TC | CC |  |  | GG | AG | AA |  |
| *Insulinogenic index* | (n=160) | (n=234) | (n=81) | MALES |  | (n=98) | (n=230) | (n=147) |  |
| Equal weight | 6.2 ± 3.8 | 5.4 ± 3.4 | 5 ± 3.4 | **0.017**/0.136 |  | 5.2 ± 2.8 | 5.8 ± 3.9 | 5.5 ± 3.5 | 0.260/NS |
| Dominant model | 6.2 ± 3.8 | 5.3 ± 3.4 | | **0.003*/0.024** |  | 5.2 ± 2.8 | 5.7 ± 3.7 | | 0.438*/NS |
| Recessive model | 5.7 ± 3.6 | | 5 ± 3.4 | 0.136*/NS |  | 5.6 ± 3.6 | | 5.5 ± 3.5 | 0.746*/NS |
|  |  |  |  |  |  |  |  |  |  |
| *2h FFA levels (mmol/l)* | |  |  |  |  |  |  |  |  |
| Equal weight | 268 ± 189 | 239 ± 96 | 215 ± 102 | **0.012**/0.096 |  | 235 ± 92 | 243 ± 163 | 254 ± 114 | 0.566/NS |
| Dominant model | 268 ± 189 | 233 ± 98 | | **0.018***/0.144 |  | 235 ± 92 | 247 ± 146 | | 0.593*/NS |
| Recessive model | 251 ± 142 | | 215 ± 102 | **0.038***/0.304 |  | 240 ± 145 | | 254 ± 114 | 0.104*/0.832 |
| *Insulinogenic index* | (n=167) | (n=269) | (n=100) | FEMALES |  | (n=117) | (n=280) | (n=139) |  |
| Equal weight | 6.3 ± 4.6 | 6.1 ± 4.5 | 5.5 ± 3 | 0.340/NS |  | 6.6 ± 4.8 | 6.3 ± 4.6 | 5.2 ± 2.9 | **0.013**/0.104 |
| Dominant model | 6.3 ± 4.6 | 5.9 ± 4.1 | | 0.372*/NS |  | 6.6 ± 4.8 | 5.9 ± 4.2 | | 0.471*/NS |
| Recessive model | 6.2 ± 4.5 | | 5.5 ± 3 | 0.616*/NS |  | 6.4 ± 4.7 | | 5.2 ± 2.9 | **0.004*/0.032** |
|  |  |  |  |  |  |  |  |  |  |
| *2h FFA levels (mmol/l)* | |  |  |  |  |  |  |  |  |
| Equal weight | 222 ± 88 | 215 ± 93 | 211 ± 92 | 0.601/NS |  | 210 ± 92 | 219 ± 86 | 217 ± 99 | 0.677/NS |
| Dominant model | 222 ± 88 | 214 ± 92 | | 0.144*/NS |  | 210 ± 92 | 218 ± 91 | | 0.434*/NS |
| Recessive model | 218 ± 91 | | 211 ± 92 | 0.316*/NS |  | 216 ± 88 | | 217 ± 99 | 0.575*/NS |
